# Application of principal component analysis to multispectral-multimodal optical image analysis for malaria diagnostics

**DOI:** 10.1186/1475-2875-13-485

**Published:** 2014-12-11

**Authors:** Dickson L Omucheni, Kenneth A Kaduki, Wallace D Bulimo, Hudson K Angeyo

**Affiliations:** Department of Physics, University of Nairobi, Nairobi, Kenya; Department of Biochemistry, University of Nairobi, Nairobi, Kenya

**Keywords:** Malaria diagnostics, Multispectral imaging, Principal component analysis, LED microscope

## Abstract

**Background:**

Multispectral imaging microscopy is a novel microscopic technique that integrates spectroscopy with optical imaging to record both spectral and spatial information of a specimen. This enables acquisition of a large and more informative dataset than is achievable in conventional optical microscopy. However, such data are characterized by high signal correlation and are difficult to interpret using univariate data analysis techniques.

**Methods:**

In this work, the development and application of a novel method which uses principal component analysis (PCA) in the processing of spectral images obtained from a simple multispectral-multimodal imaging microscope to detect *Plasmodium* parasites in unstained thin blood smear for malaria diagnostics is reported. The optical microscope used in this work has been modified by replacing the broadband light source (tungsten halogen lamp) with a set of light emitting diodes (LEDs) emitting thirteen different wavelengths of monochromatic light in the UV–vis-NIR range. The LEDs are activated sequentially to illuminate same spot of the unstained thin blood smears on glass slides, and grey level images are recorded at each wavelength. PCA was used to perform data dimensionality reduction and to enhance score images for visualization as well as for feature extraction through clusters in score space.

**Results:**

Using this approach, haemozoin was uniquely distinguished from haemoglobin in unstained thin blood smears on glass slides and the 590–700 spectral range identified as an important band for optical imaging of haemozoin as a biomarker for malaria diagnosis.

**Conclusion:**

This work is of great significance in reducing the time spent on staining malaria specimens and thus drastically reducing diagnosis time duration. The approach has the potential of replacing a trained human eye with a trained computerized vision system for malaria parasite blood screening.

## Background

Multispectral imaging is a novel imaging technology that integrates spatial and spectral information of an object under observation. Its use has been reported in food quality analysis [1–4], medical applications [5, 6] and remote sensing [7], among other applications. A multispectral image consists of a set of congruent grey-level images, each acquired at different wavelengths of light. The values of the grey levels (the recorded intensity value in each pixel) represent a measured intensity of the radiation used to capture the image. The number of grey level (intensity) images generally varies from four to a few tens depending on the spectral resolution required or equipment limitations. A typical multispectral image may consist of 512×512 pixels in the spatial domain, with each pixel (actually a voxel) consisting of a measurement spectrum for each of the wavelengths at which grey level imaging was done. As such, the amount of data obtained from a multispectral image is very large and may consist of correlated spectra. Multivariate chemometric tools such as principal component analysis (PCA) are handy in analysing such data because they search for latent structure in the raw data that best describe the measured variables to give desired information.

### Malaria diagnostics

A chemometric-based method towards rapid malaria diagnosis in unstained thin blood smear was sought for development. Malaria is a serious, infectious disease caused by a parasitic protozoon known as *Plasmodium*. It is transmitted from one human being to another via saliva of a feeding female Anopheles mosquito. According to World Health Organization global estimates of malaria cases and deaths, an approximated 0.627 million of the 207 million people infected by the disease in the year 2012 succumbed to this menace, 90% being from Africa [8]. Four species of *Plasmodium* cause malaria in humans: *Plasmodium ovale, Plasmodium vivax, Plasmodium malariae,* and *Plasmodium falciparum*. The latter is responsible for the highest mortality.

Examination of stained blood film under a light microscope for presence of *Plasmodium* is currently the gold standard for laboratory confirmation of malaria. In this technique, peripheral blood from a finger (or the heel in young infants) is smeared on a glass slide, stained and fixed (for thin blood smear) to highlight the malaria parasites. Giemsa staining is the most commonly used method [9]. The staining process can take up to 30 minutes for thin film preparation while examination of 100 fields (a field is area that is visible in a microscope) in a thick blood smear (the film preparation method that is a little faster) has been found to miss infection by up to 20% [9]. Moreover, this method is tedious as the microscopist or the examining technician has to examine as many red blood cells as possible on a glass slide with his/her own eye. A correct diagnosis is done only after attentive and careful observation of at least 100 microscopic fields has been examined in thick blood smear, and a number of morphological characteristics have been drawn to identify the species in thin blood smear. The time spent in the staining process and manual examination of many fields is the cause of delays in releasing laboratory results in hospitals. In addition, the results are subjective and may not be reproducible depending on the expertise of the examining personnel and the time spent on examination of the specimen. These limitations experienced in conventional light microscopy have stimulated research in development of alternative diagnostic methods that could be rapid, sensitive and affordable.

Most current malaria diagnostic techniques are based on any of the following five approaches: immunochromatographic tests for malaria antigen in blood [10, 11], polymerase chain reaction (PCR) [12–14], a variety of spectroscopic means of detection of haemozoin [15–17], and automated imaging microscopy of malaria parasites’ cell morphology and colour [18–20]. These methods are either accurate but very expensive, as in the case of PCR, or cheap but inaccurate due to limited information obtained from the sample, as in the case of colour imaging microscopy. In this work, PCA was combined with multispectral and multimodal imaging to detect malaria parasites in unstained human blood media for malaria diagnostics.

## Methods

### The multispectral-multimodal imaging microscope

The imaging system used in this study was a multimodal, multispectral, light emitting diodes (LED)-illuminated imaging microscope developed by Mikkel *et al.*[21] and advanced by Aboma [22]. The equipment consists of a commercial optical microscope (Brunel compound microscope) modified by replacing the conventional illumination light (white light) with monochromatic LED lighting system. Thirteen LED illuminations centred at 375 nm, 400 nm, 435 nm, 470 nm, 525 nm, 590 nm, 625 nm, 660 nm, 700 nm, 750 nm, 810 nm, 850 nm, and 940 nm are employed. This range covers ultra-violet (UV), visible and near infra-red (NIR) regions of the electromagnetic spectrum. The LEDs are mounted in a quasi-hemispherical container made of Teflon and illuminate same spot on a 5-mm Opal light diffuser (Edmund Optics, UK) to provide uniform illumination to the sample. To achieve multispectral imaging, the LEDs are activated sequentially through a computer controlled DAQ (National Instruments Inc, UK). For each LED illumination, a grey-scale image is recorded, thus resulting in 13 grey-scale images of same spot on the specimen.

The sample is illuminated in three different modes: bright field (transmission), dark field (scattering) and reflection. For transmission measurement, the sample is illuminated by a set of LEDs located directly below the sample. In reflection mode, light reaches the sample from above via a system of optics, which includes a cassegrain objective. The cassegrain objective (×15 Reflx™®, 0.28 Numerical Aperture objective, Edmund Optics, UK) is a reflective objective that is essential for reduction of chromatic aberration in all the three modes of imaging. Dark field imaging is achieved through the use of a ring coupled on an optical fibre. The ring provides an oblique illumination that cannot be accepted by objective’s aperture, hence only light which is scattered by the specimen is accepted by the objective. An image formed in this way has bright objects superimposed upon a dark background.

**Figure 1:**
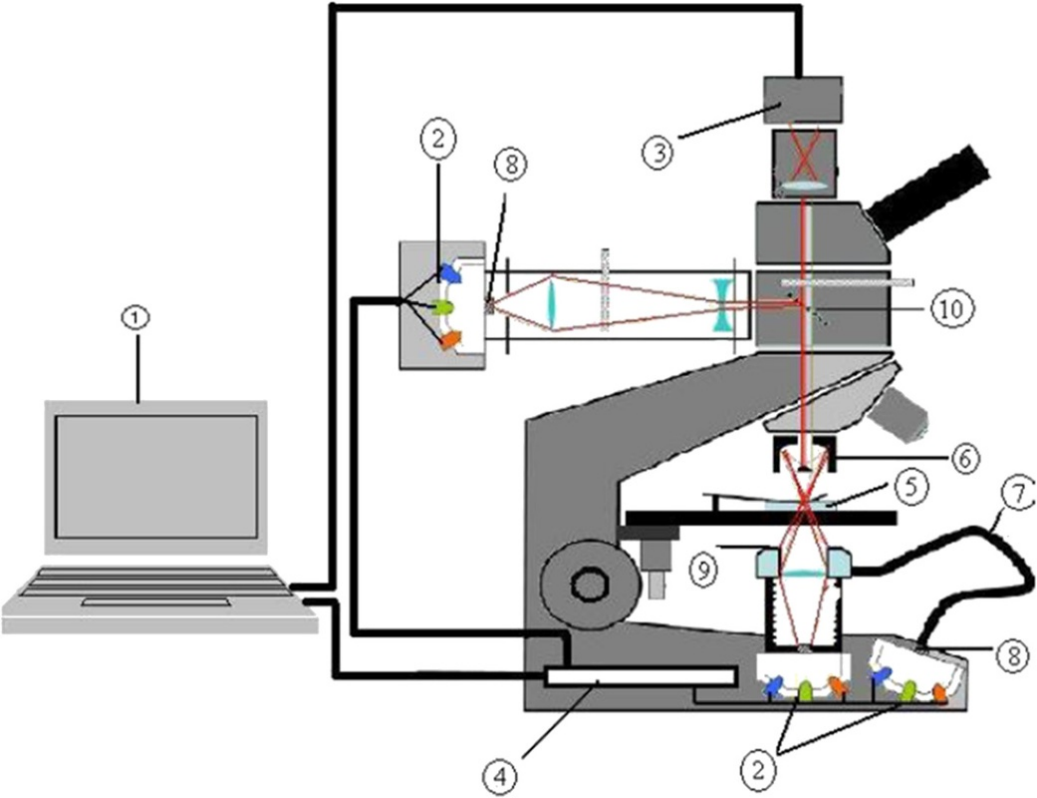
**The multispectral imaging microscope (adapted from** **[**[22]**]).** Key: 1. Computer. 2. LED set. 3. CMOS camera. 4. DAQ card. 5. Sample. 6. Cassegrain objective. 7. Optical fibre. 8. Diffuser. 9. Ring coupled on optical fibre. 10. Beam splitter.

A computer-controlled 12-bit monochrome CMOS camera (Guppy GF503B, Allied Vision Technologies, Germany) is fitted on the microscope to capture a grey-scale image of the sample at each LED illumination. With a pixel size of 2.2 μm, this camera is well within Nyquist sampling theorem and the system has a Rayleigh resolution limit 2 μm for 940 nm and 0.8 μm for 375 nm, the lowest wavelength used in the system. The system is therefore able to resolve the asexual stage of the *Plasmodium* parasite, which is about 1–2 μm. Figure 1 shows the complete imaging set-up.

### Measurement procedure

*In vitro* unstained thin blood smear malaria samples were obtained from Kenya Medical Research Institute (KEMRI). The samples had already been positively identified by an expert microscopist as infected and containing all the asexual stages of *P. falciparum*. For each sample, three measurements (transmission, reflectance and scattering) were made. For each measurement, the sample was focused such that the camera ‘sees’ the sample from all the three modes at same image plane. Since LEDs of different wavelengths have different illumination intensity and the camera quantum efficiency varies with wavelength, a protocol was created, at every measurement, in the software (developed in LabView-National Instruments Inc, UK) by adjusting the illumination intensity (current adjustment) of the LEDs, the exposure time and gain of the camera to obtain a good dynamic range of images without saturating the camera.

For each mode, three images of each wavelength were taken using the same protocol. These were the sample image, I(λ)_s_, reference image, I(λ)_r_ and dark image, I(λ)_d_. For transmission measurements, I(λ)_r_ is an image of an empty glass slide. In reflection and scattering modes, I(λ)_r_ is the image of reflecting and scattering sides respectively of an Opal diffuser (PO:006208–00 SM-Edmund Optics) known to have an average reflectance value of 50%. The dark current image I(λ)_d_ in transmission mode is taken by using the same transmission protocol as that of the sample but with the LEDs switched off. In reflection, I(λ)_d_ is obtained using the same protocol as that of the reflection sample image with nothing on the light path (sample and slide removed) while in dark field mode I(λ)_d_ is obtained with an empty glass slide using scattering sample image protocol.

### Intensity calibration

To account for the differences in the spectrum of the light sources (LEDs), the ‘dark’ camera response and different noise sources, a corrected spectral image, I(λ)_spec_ was obtained from the images captured in measurements using Equation 1.
1

where I(λ)_s_ is sample image, I(λ)_r_ reference image, and I(λ)_d_ is dark image. The subtraction is done on a pixel-by-pixel basis for each wavelength (λ). Image correction and the subsequent processing and analysis were performed using MATLAB® (MathWorks Inc, USA) software. A code was written to import the raw images stored in unsigned 16-bit representation, convert them to double class representation and apply the correction using Equation 1 in readiness for further analysis.

For images taken in transmission mode, transmittance values were obtained directly from Equation 1 since the reference was a plain transparent glass slide. The ratio between the intensity of light transmitted through sample slide to the intensity of light transmitted though an empty slide was the transmittance value. However, the values of reflectance were obtained by multiplying images taken in reflectance mode by a factor Q given by Equation (2), because the reference used in this case (Opal diffuser) is 50% reflecting.
2

Where I(λ)_d_ is the dark image normalized to unity.

## Results and discussion

### Identification of infected red blood cells in dark field

**Figure 2:**
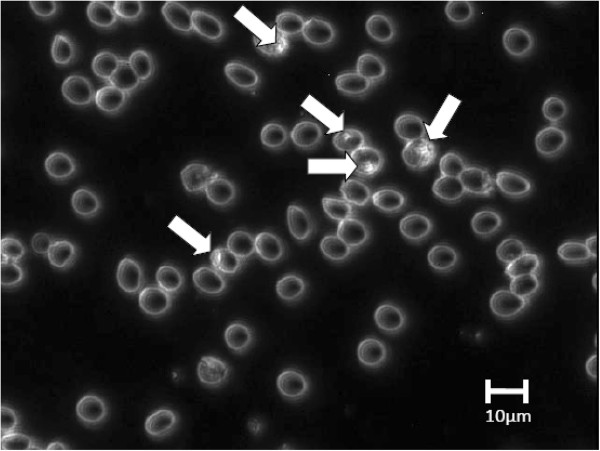
**Suspected** ***Plasmodium falciparum*** **in erythrocytes as seen in dark-field microscopic image of** ***in vitro culture*** **thin blood smear.**

Figure 2 shows dark field microscopic image of red blood cells infected with *P. falciparum*. The infected red blood cells are indicated using white arrows. Dark-field mode of imaging was used as a starting point for identifying red blood cells as either infected or not infected because parasites inside the red blood cells cause scattering which make them to be highlighted as tiny bright spots inside the red blood cells [23]. The bright (white) outline of the red blood cells is also the result of scattering of light by the edges of the red blood cells.

Since uninfected red blood cells are homogenous only their edges are seen to have scattered light. The whole scenario is observed against a dark background representing regions of little or no scattering.

However, identifying a red blood cell based on dark-field microscopic examination was not conclusive owing to the fact that morphological features well known to microscopists such as rings could not be resolved in the image. As such, it was difficult to separate a true positive from a false positive (such as an artefact, e.g., a dust particle) in the thin blood smear. Thus, a red blood cell identified as a positive in dark field had to be subjected to further scrutiny in order to arrive at a conclusion that it is a true positive. It was necessary to study the spectral dimension of images of both infected and non-infected red blood cells as identified in dark field.

### Spectral response of *Plasmodium*in blood

**Figure 3:**
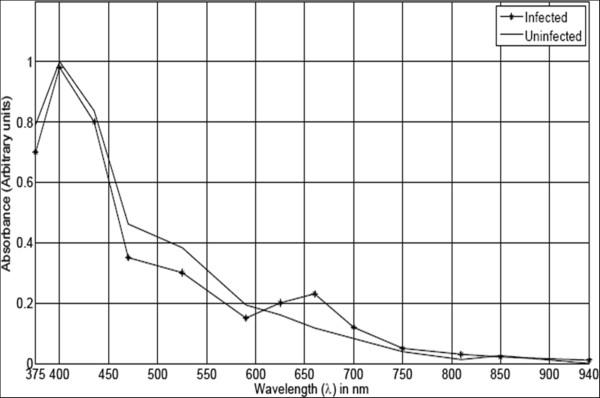
**Absorption spectra of infected and non-infected single red blood cells.**

Figure 3 shows normalized absorption spectra of *P. falciparum*-infected red blood cell and that of an uninfected one plotted together. These plots represent a trend after examining spectra of 200 non-infected red blood cells and 50 infected ones.

The spectra were obtained by cropping transmittance images of individual red blood cells (using a custom-made Matlab® function that crops a 13-band image), which gave transmittance spectra to be transformed to absorbance spectra using Equation 3.
3

where I_o_ is the incident intensity, I_t_ the transmitted intensity and A the absorbance.

It is important to note that these spectra are not absolute but only illustrate the main spectral features. The absorption spectra of infected and non-infected red blood cells have a common intense absorption band centred near 400 nm, which is due to electronic transitions of the porphyrin ring found in haem. However, there are slight differences in the spectra: a healthy red blood cell has slightly higher absorbance in the 470–590 nm wavelength range, whereas an infected one has slightly higher absorbance in the 625–700 nm spectral range.

*Plasmodium*, the malaria parasite, is a complete unicellular organism with cell membrane, nucleus and myriad organelles. On the other hand, a mature red blood cell consists mainly of haemoglobin protein without nucleus. The invasion of the red blood cell by *Plasmodium* with its cellular complexity presents some spectral response that requires a biochemical understanding of the precise origin of the spectra. Malaria parasites have nucleic acid material, which is generally transparent in the visible region. In fact, nucleic acids are identified by their characteristic intense absorption at 260 nm [24], which is outside the range of the microscope used in this work. This therefore rules out any possibility of detecting the parasites’ DNA using the multispectral microscope in its current spectral range.

An attack by blood-feeding organisms, such as malaria parasites, is associated with production of a large amount of waste product known as haemozoin (also known as malaria pigment). Haemozoin is a haem crystal produced by *Plasmodium* in order to detoxify free haem released when haemoglobin is digested in the digestive vacuoles of the parasites. Free haem is a potent cytotoxic agent to the parasites because it promotes lysis of the cellular organisms and generates reactive oxygen species (ROS), which damage many biomolecules [25]. Both haemoglobin and haemozoin contain tightly bound metal-ion-containing molecules known as prosthetic groups. The spectral signatures of prosthetic groups are known to be found in the visible region of the electromagnetic spectrum [24]. Haemozoin is, therefore, of central importance in identifying infected red blood cells spectroscopically in unstained thin blood smear using the LED multispectral imaging microscope working in 375–940 nm range. The slightly higher absorbance of infected red blood cell in the spectral range 625–700 nm but lower absorbance in the 470–590 nm can therefore be attributed to reduction of haemoglobin but increase in haemozoin in an infected red blood cell in the trophic stage of the parasite.

Reflectance spectra for both parasite-infected and non-infected red blood cells were also generated from reflectance images. However the differences between infected and uninfected red blood cell spectra were not reproducible, probably due to misalignment of the instrument and hence are not reported here.

### Image analysis based on multivariate chemometric techniques

The spectral data generated from the images were explored for any observable relational phenomena, such as clusters of pixels, gradients between the clusters and outliers. A good starting point was to use PCA, a technique that has been extensively employed successfully in multivariate image applications [26–31] to segment features based on their spectral characteristics.

### Segmentation using principal component analysis (PCA)

**Figure 4:**
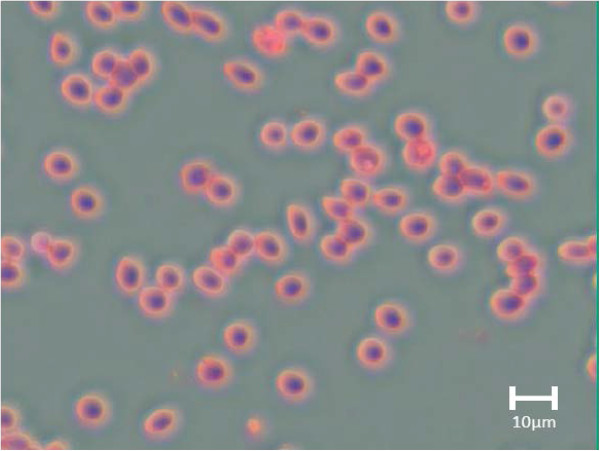
**A false-colour composite of transmittance image generated by assigning the first three principal components to red, green and blue channels.**

PCA gives two important values: scores and loadings. The role of score values in PCA image analysis is two-fold: they can be observed as both grey-level, decorrelated images and/or scatter plots. The first three score images (each of the score images is a grey-level image) which contain most of information can be assigned to red, green and blue channels to form a false RGB image that has greatest contrast since the score vectors are orthogonal to each other. In addition, the scores can be visualized as score plots and segmentation done by studying the density of pixel clusters. Figure 4 shows a false colour composite of transmittance image generated by assigning the first, second and third principal components (the first three PCs contain 99% variance) to red, green and blue channels, respectively.

In the RGB image (Figure 4), any reddish, greenish or bluish colours observed signify the first, second and third principal components, respectively. It can be seen that all the red blood cells in the image appear reddish, implying high values of the red channel (first principal component) in the red blood cells. This means that the first principal component represents haemoglobin, a dominant pigment in the red blood cells. The image background appears greyish-green, a colour that arises from slightly higher values of the green channel (second principal component) than the other channels in this region of the image. The second principal component therefore represents the image background, which is majorly blood plasma. The little bluish colour on the red blood cells in the image is due to intra-cell variance resulting from curvature of the red blood cells, an implication that the third principal component has little significance in specific features in the whole image.

Visualizing score images as colour images is a rather subjective exercise because there may be differences in screen settings and people usually perceive colours differently. A more objective way is to study the principal components as score plots. The score plots generate clusters based on the fact that similar spectral features in the original image yield almost identical score combinations. Therefore, pixels from a specific feature in the image will overlap to create a dense cluster. These clusters are of great use in segmenting features in the image by delineating pixel clusters in the score plot, which have similar spectral fingerprints. Pixel delineation is achieved by toggling between the score space and the image space. A cluster in the score space is selected and their original spatial location in the image space highlighted based on a threshold value in a grey level image calculated using Equation 44

where h1 is a grey-level image upon which thresholding is done, PC1 and PC2 are the scatter-plotted principal components. This procedure of calculating *h1* is synonymous to that of calculating normalized difference vegetation index (NDVI) in satellite images [32]. In order to extend this to the 3^rd^ PC, an additional image h2 is used to threshold PC3 from PC2 as shown in equation 5.
5

**Figure 5:**
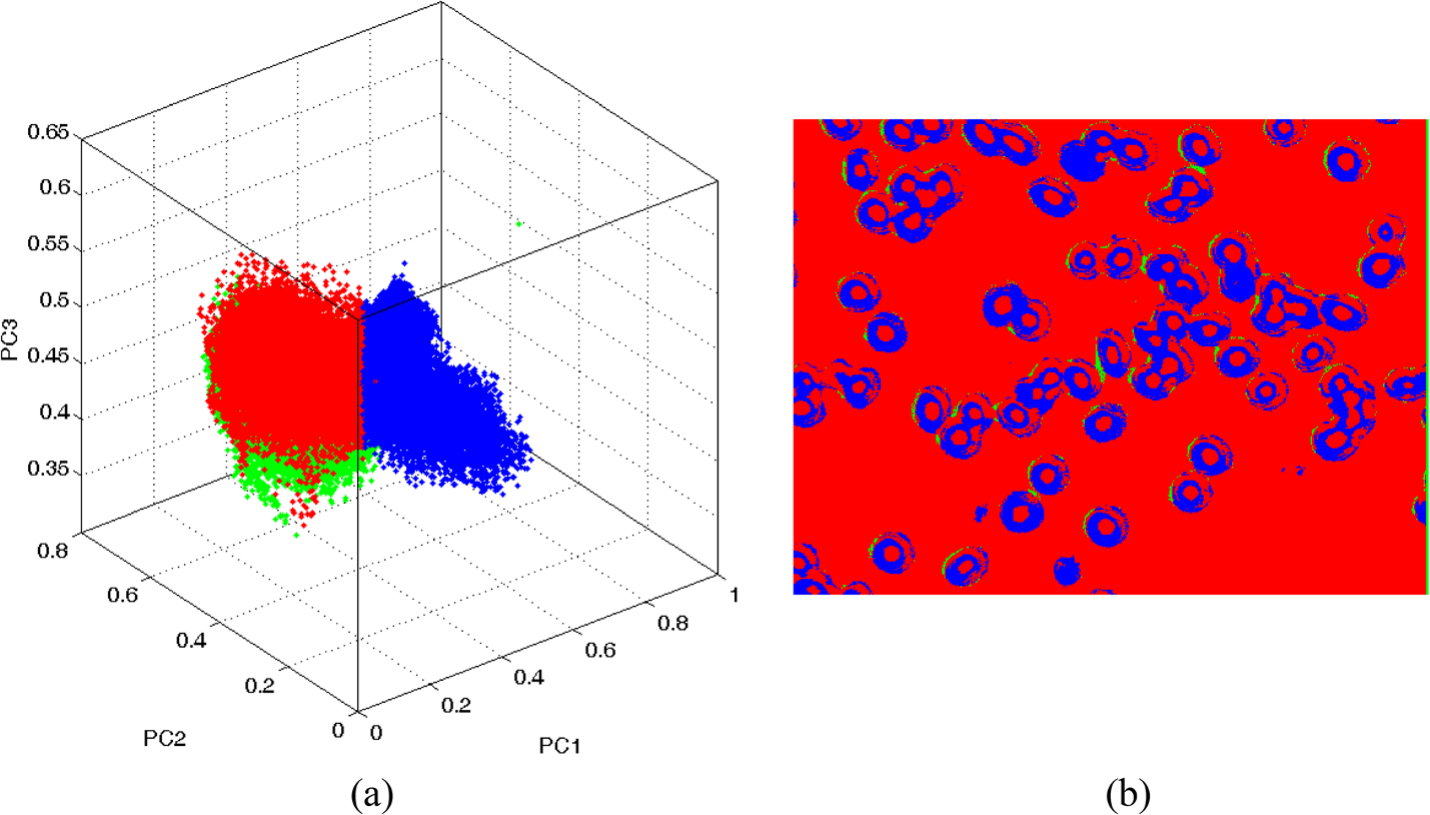
**Pixels of red blood cells for a transmission image (colour coded-blue) (a) delineated in the score space and (b) highlighted in the image space.**

Pixels falling below the threshold can be isolated and projected back in the image. By varying the threshold value of *h1* and *h2,* specific features in the image can be highlighted including the parasite. Figure 5 shows clusters in the 3D score space and their corresponding origin in the image space.

**Figure 6:**
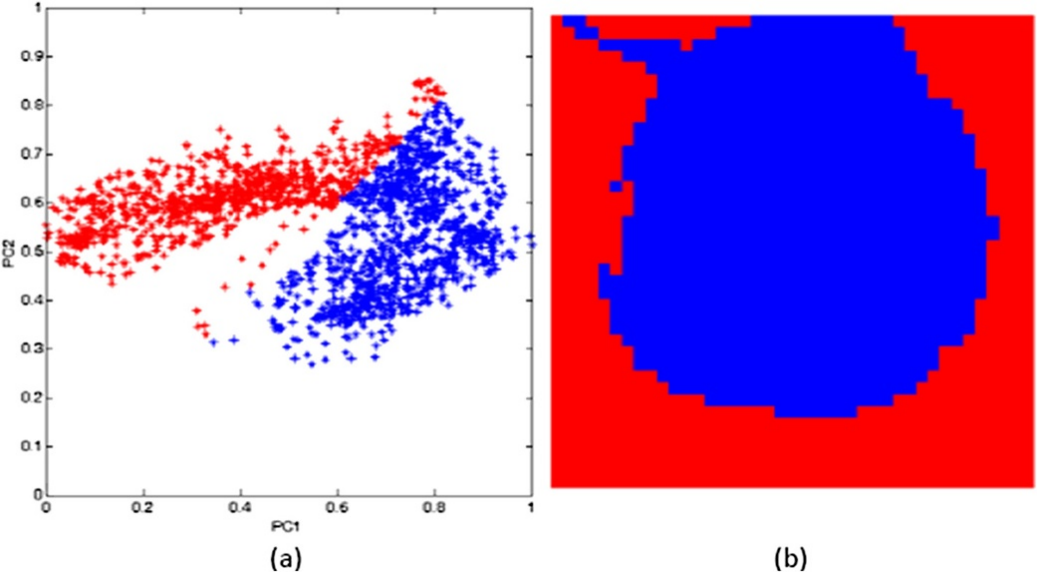
**Pixels of a single red blood cell (colour coded-blue) (a) delineated in the score space and (b) highlighted in the image space.**

**Figure 7:**
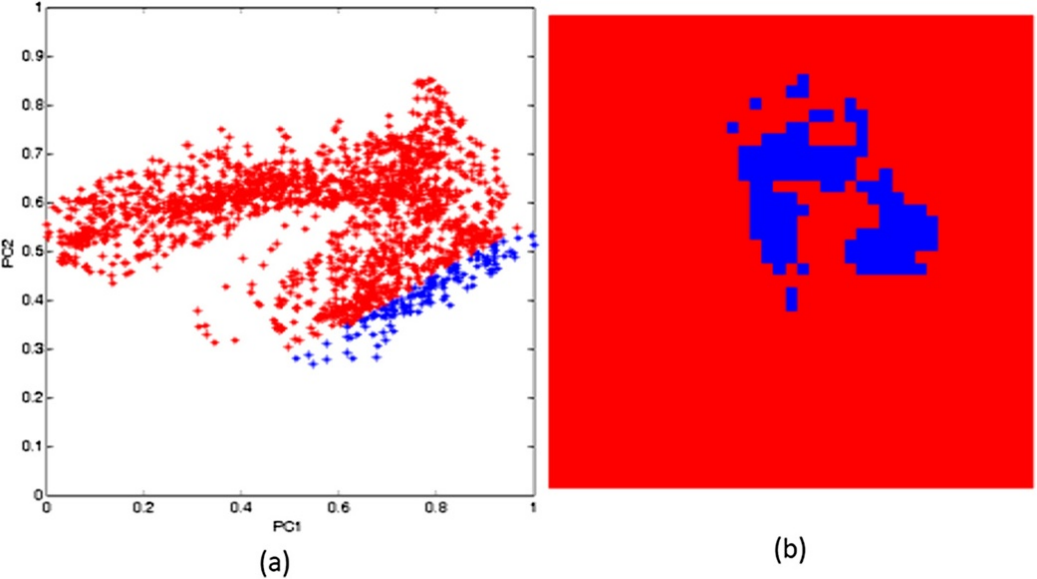
**Pixels of a malaria parasite (colour coded-blue) (a) delineated in the score space and (b) highlighted in the image space.**

It is clear that the pixels in the blue cluster originated from the red blood cells. However, highlighting malaria parasites inside the red blood cells at this level may be a daunting task as the pixels from the parasites form a very small cluster overlapping with those of the red blood cells. As such, individual single red blood cells were cropped and the process of delineating pixels in the score space and highlighting them in the image space repeated. The results of this process are shown in Figures 6 and 7.

From Figures 5, 6 and 7, it can be seen that the pixels from the parasite form a small cluster within the pixel cluster of the red blood cell. Overlapping of these clusters may have arisen from the fact that pixels in an infected red blood cell image contain mixed spectra of haemoglobin and haemozoin. In addition, the spectral features of haemozoin and haemoglobin are highly correlated. Both haemoglobin and haemozoin contain haem (a prosthetic group), which exists as a large heterocyclic organic ring called porphyrin with an iron ion at the centre. In haemozoin, this ring exists as ferriprotoporphyrin IX [33] whereas the non-protein part of haemoglobin exists as ferroprotoporphyrin IX. These two compounds exhibit π-π* electronic transitions of the porphyrin ring visualized as the strong absorption band (Soret band) centred near 400 nm as seen in Figure 3. Their differences, however, arise from different locations of their vibronic transitions which occur at 540 nm and 575 nm for oxy-haemoglobin and 650 nm for haemozoin.

### Important spectral band selection

**Figure 8:**
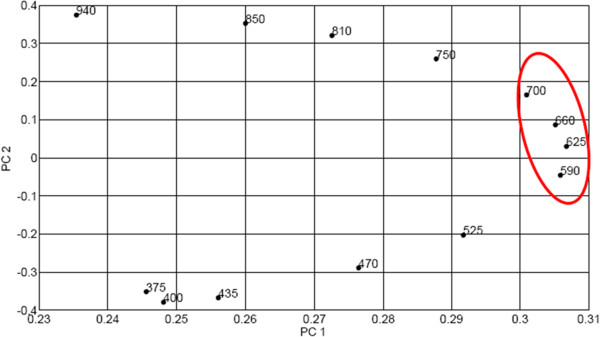
**Loading plot of PC2 against PC1 from transmission image.** The loadings represent the weight of each PC at the specified wavelengths.

The influence a measured variable has in each score is given by its loading. Hence, the loading plots are helpful in understanding the meaning of groups (in this case pixel clusters). Variables responsible for higher variance in the datasets generally give higher coefficients (positive or negative) on a certain principal component. In this regard, the contribution from different spectral bands to the principal component eigen vectors can be observed in the loading plots. Figure 8 shows the loading plot of the first two principal components of images captured in the transmission mode.

It was observed that the loading plots of the first and the second principal components of transmittance images were quite systematic for different red blood cells. From Figure 8, it can be observed that the variables loading heavily on the first principal component are 590 nm, 625 nm, 660 nm, and 700 nm. These are bands associated with the vibronic absorption bands of haemoglobin and haemozoin. They are the important bands for differentiating between infected and uninfected red blood cells. Along the second principal component, infrared bands (750–940 nm) have high positive loading values whereas ultraviolet (i.e., 375 nm) and blue region of visible light (400–525 nm) have high negative values. It is a clear indication of contrasting absorption behaviour of haemoglobin in the long wavelength (IR) radiations and short wavelength (UV and blue part of visible light) of the optical spectrum. In the former, haemoglobin exhibits very little (or no absorption) whereas in the latter, it absorbs strongly. This is also the case with haemozoin. Since haemoglobin and haemozoin have identical optical characteristics in these two spectral regions, it implies that detection of malaria parasites can be done by employing fewer LEDs emitting in the 590–700 nm spectral range in the multispectral microscope.

### Image generation by selective illumination of red blood cells

**Figure 9:**
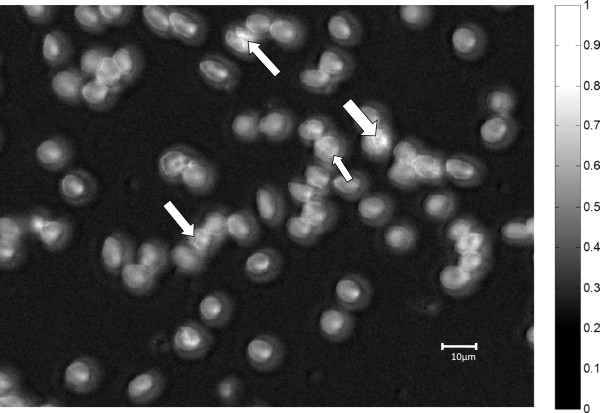
**Image captured at a common absorption band (400 nm) for haemozoin and haemoglobin.**

**Figure 10:**
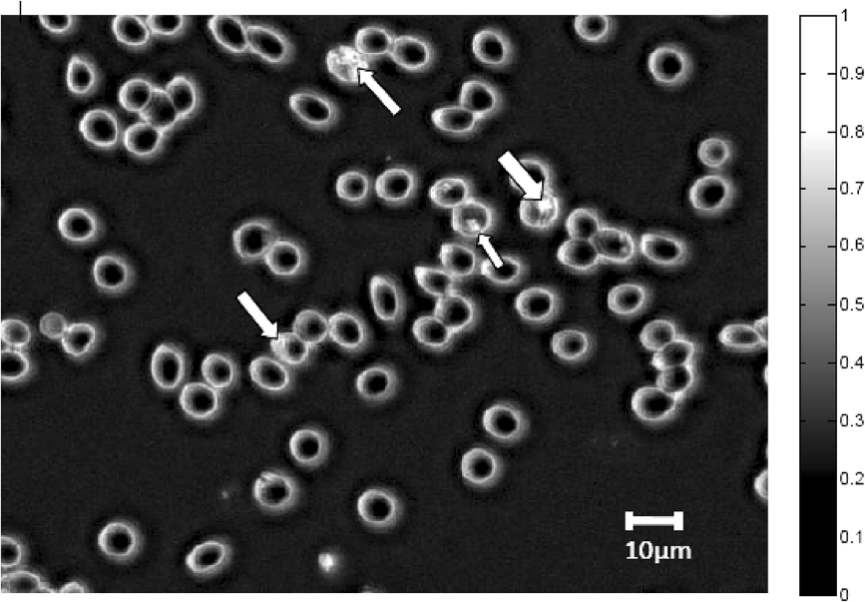
**Grey-level image captured at 625 nm showing high absorption values for parasite region (shown with white arrows).**

**Figure 11:**
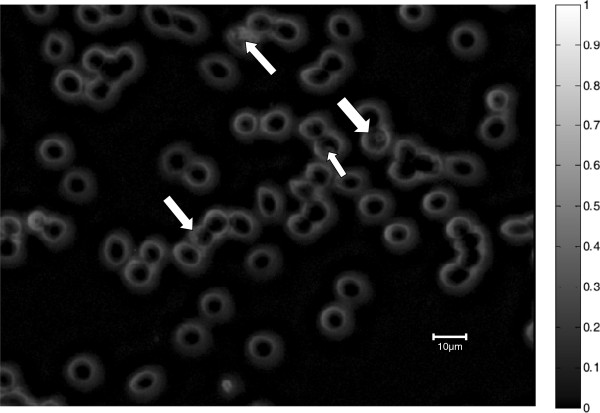
**Image captured at a common non-absorbing band (940 nm) for haemozoin and haemoglobin.**

After PCA results had shown that the variables (wavelengths) of importance in malaria diagnostics for the microscope were 590–700 nm, raw grey-level transmittance images captured at these important variables (wavelengths) were transformed to absorbance (by use of Beer-Lambert’s law as explained above) and compared to images captured at wavelengths contributing little variance such as those captured at 375 nm, 400 nm, 850 nm, and 940 nm. Figures 9, 10 and 11 show grey-level absorbance images captured at 400 nm, 625 nm and 940 nm, respectively.

In the image captured at 625 nm (Figure 10), the parasite region appears brighter due to its higher absorption in the image in comparison with other parts of red blood cells (see the grey-level scale). However, in the image captured at 400 nm (Figure 9) and that captured at 940 nm (Figure 11), there is little intensity difference between the parasites and the red blood cells. The reason for this is that at 400 nm and at 940 nm, the malaria pigment has almost identical absorption properties to those of haemoglobin. At 625 nm (and at the other three important spectral bands *viz* 590 nm, 660 nm and 700 nm) however, there are slight differences in absorption of haemoglobin and haemozoin, making the parasite more visible.

These results are in agreement the findings of Aboma Merdasa *et al*. [34]. According these findings, the greatest difference between infected and uninfected red blood cells when analyzed using singular value decomposition and hierarchical clustering for all the three modes of imaging is found at around 630 nm.

## Conclusion

The application of PCA to multispectral image analysis has been demonstrated. The spectral images were recorded using a simple LED-illuminated microscope equipped with a CMOS camera. Using PCA malaria parasites in infected red blood cells were successfully segmented and important spectral bands for optical imaging of malaria parasites in unstained thin blood smear identified. This is of great significance in reducing the time spent on staining malaria specimens and thus drastically reducing diagnosis time duration.
